# Computational and Experimental Study of Nonlinear Optical Susceptibilities of Composite Materials Based on PVK Polymer Matrix and Benzonitrile Derivatives

**DOI:** 10.3390/ma15062073

**Published:** 2022-03-11

**Authors:** Lucia Mydlova, Bouchta Sahraoui, Karolina Waszkowska, Houda El Karout, Malgorzata Makowska-Janusik, Anna Migalska-Zalas

**Affiliations:** 1 Faculty of Science and Technology, Jan Dlugosz University, Al. Armii Krajowej 13/15, 42-200 Czestochowa, Poland; lucia.mydlova@ujd.edu.pl; 2 Laboratoire MOLTECH Anjou, Université d’Angers, UFR Sciences, UMR 6200, CNRS, Bât. K, 2 Bd. Lavoisier, CEDEX 01, 49045 Angers, France; bouchta.sahraoui@univ-angers.fr (B.S.); vaszkowska@gmail.com (K.W.); houda.elkarout@univ-angers.fr (H.E.K.)

**Keywords:** nonlinear optics, guest–host composite, hyperpolarizability, density functional thery (DFT), molecular dynamics, nonlinear optics (NLO), PVK

## Abstract

Theoretical and experimental investigations of the linear and nonlinear optical properties of composite materials based on the (Z)-4-(1-cyano-2-(5-methylfuran-2-yl)vinyl)benzonitrile molecule named as A, the (Z)-4-(2-(benzofuran-2-yl)-1-cyanovinyl)benzonitrile named as B and the (Z)-4-(2-(4-(9H-carbazol-9-yl)phenyl)-1-cyanovinyl)benzonitrile molecule named as C embedded into poly(1-vinylcarbazole) (PVK) polymer matrix were performed. The electronic and optical properties of A, B, and C molecules in a vacuum and PVK were calculated. The guest–host polymer structures for A, B, and C molecules in PVK were modeled using molecular dynamics simulations. The spatial distribution of chromophores in the polymer matrix was investigated using the intermolecular radial distribution (RDF) function. The reorientation of A, B, and C molecules under the influence of the external electric field was investigated by measuring the time-dependent arrangement of the angle between the dipole moment of the chromophore and the external electric field. The polarizabilities and hyperpolarizabilities of tested compounds have been calculated applying the DFT/B3LYP functional. The second- and third-order nonlinear optical properties of the molecule/PVK thin film guest–host systems were investigated by the Maker fringes technique in the picosecond regime at the fundamental wavelength of 1064 nm. The experimental results were confirmed and explained with theoretical simulations and were found to be in good agreement. The modeling of the composites in volumetric and thin-film form explains the poling phenomena caused by the external electric field occurring with the confinement effect.

## 1. Introduction

Organic chromophores are still intensively investigated because they have widespread application in high-speed electro-optic devices as core components of electro-optically active materials due to their large bandwidths and low drive voltages [1,2,3]. Interesting organic chromophores for optical applications are A-π-D-type molecules expected to show large nonlinear optical (NLO) responses. Typically, they contain a conjugated π-electron system, asymmetrically substituted by electron donor (D) and acceptor (A) groups. Such systems can be described by one dominant hyperpolarizability component lying in the direction of charge transfer.

For practical optoelectronic applications, the NLO chromophores should be incorporated into a macroscopic environment, and this can be carried out in a variety of ways. One of the most widely used is the incorporation of chromophores into an amorphous polymer host by simply dissolving the chromophores into a polymeric material (guest–host system), by covalently attaching the chromophores to a polymeric backbone (side-chain polymers) or by incorporating the chromophores into the backbone of the polymer (main-chain polymers) [4]. To achieve the necessary non-centrosymmetry of the material for its second-order NLO response to occur, the chromophores in the polymer matrix need to be aligned. This can be carried out by poling the dipolar chromophores with an external electric field (electric field poling) while heating the polymer to near its glass transition temperature (*T_g_*) where it becomes rubbery. Cooling the systems in the presence of the mentioned electric field freezes the orientation of the chromophores. The degree of alignment depends on several factors, e.g., structure of chromophores as well as dynamic and structural properties of the polymeric matrix [5].

**Figure 1:**
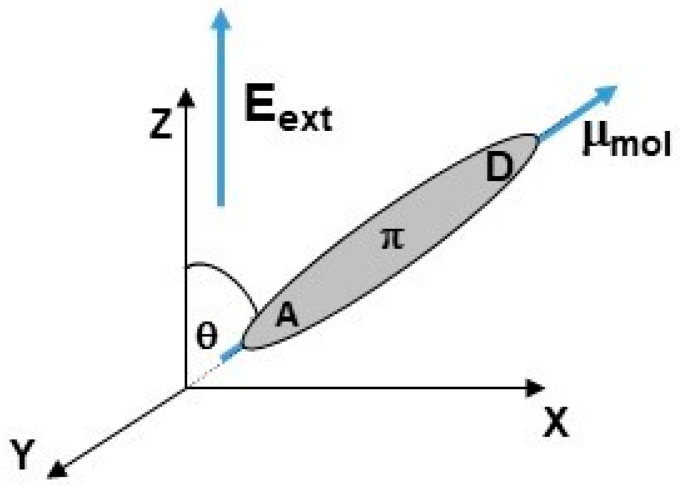
The location of the θ angle defines the position between the direction of the external electric field (E_ext_) and the electric dipole moment (μ_mol_) of a chromophore molecule. The molecule is presented in a laboratory coordinate system.

Generally, an external electric field (E_ext_) is applied to regulate individual chromophore alignment. The permanent electric dipole moment of each chromophore acts with the poling electric field and induces a partial alignment of the dipoles, whose average projection in the direction of the poling field can be characterized by <*cos θ*>. The *θ* is the angle between the direction of the electric dipole moment vector of the chromophore and the external electric field (see Figure 1). The parameters <*cos θ*> can be studied by dipolar orientation dynamics of chromophores and their relaxation behavior versus time.

Simulations of guest–host composite systems based on small chromophores meet with the aggregation or association effects involving the condensation of molecules onto a surface, phase changes of the physical state of matter, or phase-separation phenomena [6]. The orientational effects associated with the interactions between chromophores and the host matrix have a huge impact on the local field (*F*) felt by the chromophores and also consequently affect the degree of their arrangement in the self-consistent structural rearrangement process [7]. This structural question is quite interesting of determining how to reach the non-centrosymmetric arrangement of chromophores without their back-relaxation to the random orientational distribution [8]. The efficiency of the alignment of the chromophores in the polymer matrix also depends on their location; therefore, the spatial distribution of chromophores can be investigated using the molecular dynamics (MD) methodology.

Several MD simulation techniques have been denoted to investigate the behavior of NLO chromophores in the polymeric matrixes. Simulations of the poling process of organic materials with an external electric field are described in the works of Robinson [6,8] and Kim and Hayden [9]. Robinson considered the electrostatic interaction between molecules subjected to an external electric field and analyzed the influence of external fields on the mobility of molecules in non-centrosymmetric systems subjected to Monte Carlo simulations. Kim and Hayden studied the ability to organize the composite material with an external electric field by changing the density of the system, which was to imitate a composite material in a liquid and solid state of matter. In none of the works mentioned above, the phase transition of the composite material subjected to the poling process was not considered. In our previous work [10], we proposed modeling the phase transition process of the poled guest–host system using the MD. The proposed methodology was implemented for volumetric composite materials. The composites were polarized by the external electric field (E_ext_) in the liquid state, and then the process of gradual transition of the system from the liquid to the glass state was simulated maintaining the poling order of the chromophores. Over the past few years, according to the practical application of composite materials, polymer films have received considerable attention. The polymer thin film exhibits thermodynamic, structural, and dynamic properties different from those of the bulk material [11]. The surface properties of polymers are a critical factor in determining performance and suitability in films and coatings. Numerical simulations can help to understand the surface mobility of chromophores and explain the NLO properties of composites. The main assumption of the presented work was to model the non-centrosymmetric material in the volumetric and thin-film form, used for the generation of NLO effects and to check the effect of material structure on its optical properties [12].

The aim of this study is to carry out theoretical and experimental investigations of the optical linear and NLO properties of composite materials based on the (Z)-4-(1-cyano-2-(5-methylfuran-2-yl)vinyl)benzonitrile molecule named as A, the (Z)-4-(2-(benzofuran-2-yl)-1-cyanovinyl)benzonitrile named as B and the (Z)-4-(2-(4-(9H-carbazol-9-yl)phenyl)-1-cyanovinyl)benzonitrile molecule named as C embedded into poly(1-vinylcarbazole) (PVK) polymer matrix in volumetric and thin film form. The findings presented below follow a previously published article, in which the structural and electronic properties of the mentioned A, B, and C molecules were described [13]. The A, B, and C are benzonitrile moiety-based molecules. The benzonitrile is commonly used as a functional group in NLO chromophores [14], acting as an electro-acceptor [15,16]. In the present work, we would like to explain the nature of the NLO properties of the A/PVK, B/PVK, and C/PVK composites. We present methodology on how to predict the NLO properties of the thin films of composites using MD simulations and quantum chemical calculations.

## 2. Materials and Methods

### 2.1. Computational Simulations

**Figure 2:**
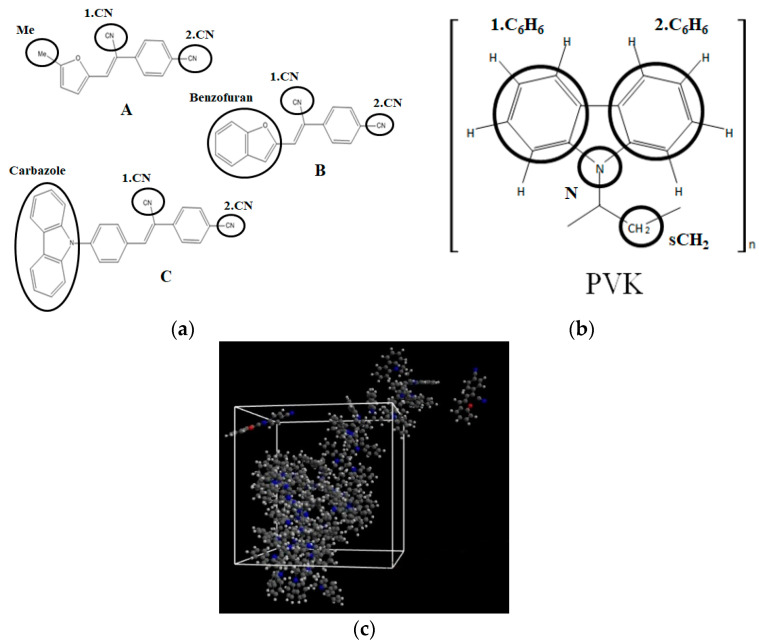
Structures of investigated chromophores A, B, C (**a**), mer of the PVK polymer (**b**) with marked their atomic groups chosen for the RDFs calculations and the structure of the B/PVK system (**c**) as an example of investigated composites.

Theoretical calculations of the electronic and optical properties of the A, B, and C molecules (see Figure 2a) start with the procedure of their structure optimization. Their geometries were optimized applying the ab initio Hartree–Fock (HF) formalism implemented in the Gamess program package [17,18]. The procedure has been performed for isolated structures in a vacuum. The minimum of the potential energy surface was computed at the restricted HF (RHF) [19] level with the 6-311G basis set in C1 symmetry. The gradient convergence criterion was equal to 10^−6^ Hartree/Bohr. Geometries of the investigated molecules were found applying the quadratic approximation (QA) optimization algorithm [20] based on augmented Hessian techniques.

The electronic and optical properties of the A, B, and C molecules in a vacuum and PVK polymer matrix were calculated. The electronic properties of the mentioned molecules with optimized geometries as well as their polarizabilities *α* and hyperpolarizabilities *β* and *γ* were calculated by the time-dependent density functional theory (TDDFT) method, applying the B3LYP functional according to our previously performed calculations [13]. The calculations were performed at the RHF level with the standard 6-31++G** basis set in C1 symmetry for all chromophores. Frequency-dependent polarizabilities and hyperpolarizabilities were computed with the random phase approximation (RPA), using the Dalton program package (Dalton2020.0.beta (2020), http://daltonprogram.org, accessed on 6 March 2022) [21].

The guest–host structures for the **A**, **B**, and **C** molecules embedded into PVK polymer were built using MD simulations implemented in GROMACS 5.0.4 software (https://www.gromacs.org/, accessed on 6 March 2022)) [22]. Three initial complexes based on two chromophore molecules and one polymer chain, closed in one unit cell, were created for each **A**/PVK, **B**/PVK, and **C**/PVK composite system. The polymer was built as one isotactic 90 mer PVK chain with a molecular weight of 9012.58 amu. The structures of chromophores and mer of PVK are presented in Figure 2a,b, respectively. Each investigated unit cell was cubic with an edge length equal to 25.40 Å. The corresponding density was equal to 0.98 g/cm^3^ for **A**/PVK (5.5 wt% of the **A** molecules in the system), 1.0 g/cm^3^ for **B**/PVK (5.8 wt%), and **C**/PVK (6.3 wt%). An example of the composite system is presented in Figure 2c. The obtained densities correspond to the liquid state of the investigated complexes. Each initial composite structure has been optimized by two methods. Firstly, the steepest descent algorithm was used with a convergence criterion equal to 200 kcal mol^−1^ Å^−1^. Then, the optimization procedure was continued by the conjugate gradient method with a convergence criterion equal to 50 kcal mol^−1^ Å^−1^. The MD simulations were performed in the x,y,z periodic boundary conditions.

The MD based on solving the classical equations of motion for the time-dependent positions (and velocities) of atoms comprising the system under investigation was performed. The potential energy was computed as the contribution of all bond bending, bond stretching, dihedral angle torsion, and nonbonded interactions:

(1) $$\begin{array}{c} U=\sum_{all\;bonds}U_{bond}\left(l_{i}\right)+\sum_{all\;angles}U_{angle}\left(\theta_{i}\right)+\\ \sum_{all\;dihedral\;angles}U_{torsional}\left(\phi_{i}\right)+\sum_{all\;pairs\;i,\;j}U_{nonbonded}\;\left(r_{ij}\right) \end{array}$$

where $l_{i}$, $\theta_{i}$ and $\phi_{i}$ refer to bond length, bond angle, and dihedral angle, respectively, and $r_{ij}$ is the distance between atoms *i* and *j*. The nonbonded interactions were accounted with the Lennard-Jones 12-6 potential. The Coulombic potential was calculated using fixed partial charges of atoms including the interaction of the external electric field. Bonded interactions are computed due to a fixed list of atoms. Bond stretching and bond bending are described by harmonic potentials, whereas dihedral angle distortions are modeled by a simple cosine function. The all-atom consistent valence force field (CVFF) [23,24] was selected for the presented MD study.

All MD simulations in this work were employed with the following parameters: simulation time step was 1 fs, the short-range neighbor list was created employing the grid search method with cut-off distance 1.00 nm. Lennard-Jones and Coulomb interactions were computed within the neighbor list employing periodic boundary conditions. The long-range interactions were calculated using the three-dimensional particle-mesh Ewald method (PME) [25,26]. The cut-off distance was chosen to be equal to 1.1 nm and this was the same for all complexes. The energies, coordinates, and velocities were recorded every 1 ps. Most of the simulations were performed in the canonical NVT ensemble using the extended Nosé-Hoover algorithm. Additionally, simulations in an NPT ensemble were performed applied the Rahman–Parrinello method to control the pressure during simulations.

First, the geometry-optimized composite structures were relaxed for 2.0 ns at 500 K to obtain their equilibrium state. The stable total energy was reached after 1.5 ns of MD simulations for all structures. The next step of the MD simulations was devoted to aligning the chromophores in a polymeric matrix above the *T_g_* temperature. In this case, the poling external electric field in the Z-direction to all systems was applied and the structures were modeled for 1.5 ns. The intensity of the applied electric field for bulk complexes was equal to 1, 3, 5, 10, or 15 kV/μm. One should note that the experimentally commonly used electric field in the corona poling process is equal to 5 kV; however, we want to achieve the steady state of the composite system within the time scale of the simulations, why the used electric field intensity should be correspondingly higher.

Our previous studies [10] have proved that when using the CVFF force field, the *T_g_* of the poly(methyl methacrylate) polymer matrix (PMMA) with embedded chromophores is lower than 500 K. Experimentally, it was shown that the *T_g_* of the PMMA is 378–438 K [27]. Experimentally measured *T_g_* of the PVK is equal to 435 K [28]. In this case, it was decided that the PVK polymer with small chromophores in its matrix can be modeled at 500 K as in the liquid state and 300 K as in the glassy solid state. Based on these results, we assumed that the simulated structures at 500 K are a slightly above the glass transition temperature with the chromophores being able to be rotated according to the application of the external electric field. To model a solid-state structure representing the experimentally used guest–host materials, the simulated annealing (SA) process was implemented for all investigated systems. In this case, the simulations were performed in NPT condition for 1.5 ns, with the cooling of the system from 500 K up to 300 K. During the simulation, the external electric field was acted on the sample. The cooling rate, in our case, was 1.3 × 10^11^ K/s.

The SA is conveniently used as an optimization technique, mainly well suited to overcome the multiple minima problem. During SA, molecules may cross barriers between conformational energy minima to achieve the lowest minimum. We have used SA with the MD algorithm in the isobaric–isothermal ensemble with a protocol implemented in GROMACS, where the reference temperature is varied linearly. After annealing, the simulated systems were relaxed for 1.5 ns in the NVT assemble without an external electric field. As a consequence, the stability and back-relaxation of the chromophores were investigated.

### 2.2. Simulation Methodology of Thin Films

Three different thin film structures were created from bulk A/PVK, B/PVK, and C/PVK composite systems. Composite materials consist of one isotactic 90 mer PVK and two molecules—A, B, or C—as it was in the case of bulk materials. The simulation box of the investigated thin film system was equal to 26.88 Å × 26.88 Å × 80.64 Å. The two equivalent structures were simulated for each thin film system. The change in cubic cell of the bulk materials to the thin film unit cell as well as all molecular simulations were performed using the GROMACS 5.0.4 software package. The geometry of each thin film composite system was optimized according to the total energy minimization by the steepest descent method applying a convergence criterion equal to 100 kcal mol^−1^ Å^−1^ and then by the conjugate gradient method with a convergence criterion of 20 kcal mol^−1^ Å^−1^, followed by an NVT and NPT MD simulation. The composite systems were relaxed using the MD simulation with the time 1.5 ns at 500 K using a step size of 1 fs. The trajectories were recorded every picosecond. The CVFF was implemented for the MD study. All considered structures were relaxed for 1.5 ns at 500 K to obtain the equilibrium state of the system.

Investigated thin film composites were simulated by MD at temperature *T* = 500 K using two-dimensional (2D) periodic boundary conditions. The long-range nonbonded interactions were calculated using the 2D Ewald method, to simulate the condition of the thin film. Infinite extensions of the composite systems have been applied along the X and Y axes and the finite length in the Z-direction was defined. A constant temperature during simulations was achieved by the NVT canonical ensemble using the Nosé–Hoover method, and constant pressure was maintained by the Parrinello–Rahman method (NPT ensemble). The obtained structures in the liquid state were exposed to a poling external electric field applied along the Z-axes and simulated for a further 1.5 ns. The values of the external poling field were equal to 0.5, 1, 3, or 5 kV/μm. Then, the thin film structures were annealed from 500 K up to 300 K, from liquid to glassy state. This was carried out in the presence of the poling electric field for 1.5 ns with a cooling rate of 1.3 × 10^11^ K/s. The final density for all thin films was 1.173 ± 0.005 g/cm^3^ and corresponds to the experimental value of the glassy state of PVK.

### 2.3. Samples Preparation

The A, B, and C molecules were dissolved in 1 mL of the 1,1,2-trichloroethane (1,1,2-TCA) with 30 mg of poly(1-vinylcarbazole) (PVK) forming 5 wt% and 10 wt% solutions. Thin films were prepared by the spin coating method on appropriately cleaned glass substrates. Photographs of prepared thin films are presented in Table 1. The thickness of the prepared thin films was measured by using a profilometer (Dektak 6M, Veeco Instruments, Inc. 2650 East Elvira Road Tucson, AZ 85706, USA). 

### 2.4. Experimental Sets

UV–Vis spectra were measured for all prepared films by using the Shimadzu UV-1800 spectrometer (Shimadzu USA Manufacturing, INC. 1900 SE 4th Ave. Canby, OR 97013, USA) in the range 250–1100 nm. The second-harmonic generation SHG and third-harmonic generation THG measurements were carried out by using the Maker fringe setup [29,30]. Samples were rotated from −60° to +60° and the intensity of the generated harmonics as a function of incident angle was measured in S- and P-polarization. No restriction of polarization has been noted for THG measurements. The fundamental wavelength of the laser beam was 1064 nm, frequency 10 Hz, pulse duration 30 ps, and energy 90 μJ.

### 2.5. Molecular Orientation

The quadratic NLO response is impossible in centrosymmetric materials when the molecules are randomly oriented inside the film. The proper arrangement of the chromophores, to break the centrosymmetry of the films, has been achieved with the corona poling technique [31]. In this case, the films were heated up to temperature a bit below the *T_g_* of the polymer and strong electric voltage (4.5 kV) was applied on two 30 μm tungsten wires, which were placed over the sample. The distance between the wire and the film located on the grounded electrode was about 1 cm. The samples have been preserved under these conditions for 15 min. While keeping the electric field constant, the temperature was slowly decreased down to room temperature, resulting in the orientation of the dipole moments and inducing a non-centrosymmetry of the sample, which is required for a second-order NLO response.

## 3. Results and Discussion

### 3.1. Structure of Composite Systems

The spatial distribution of the chromophores in the polymer matrix was examined using the intermolecular radial distribution function (RDF) defined between the center of mass (COM) of selected groups of chromophores and the selected groups of the PVK mers (see Figure 2a,b, respectively). The COM of the cyanovinyl (CN) moiety in both positions of the A, B, and C molecules was selected and named as 1.CN (CN at the side of the molecule) and 2.CN (CN at the back of the molecule). Additionally, the third active group of each chromophore was chosen. Namely, the COM of the methyl group (Me) in molecule A, benzofuran of the B molecule, and carbazole of the **C** molecule were chosen. The four COMs, namely nitrogen (N) in the middle of the carbazole group, CH_2_ at the side of the main chain of the PVK, and two benzenes, the first on the left labeled as 1.C_6_H_6_ and the second one on the right labeled as 2.C_6_H_6_, were chosen. The COMs of the different groups were computed for each snapshot.

**Figure 3:**
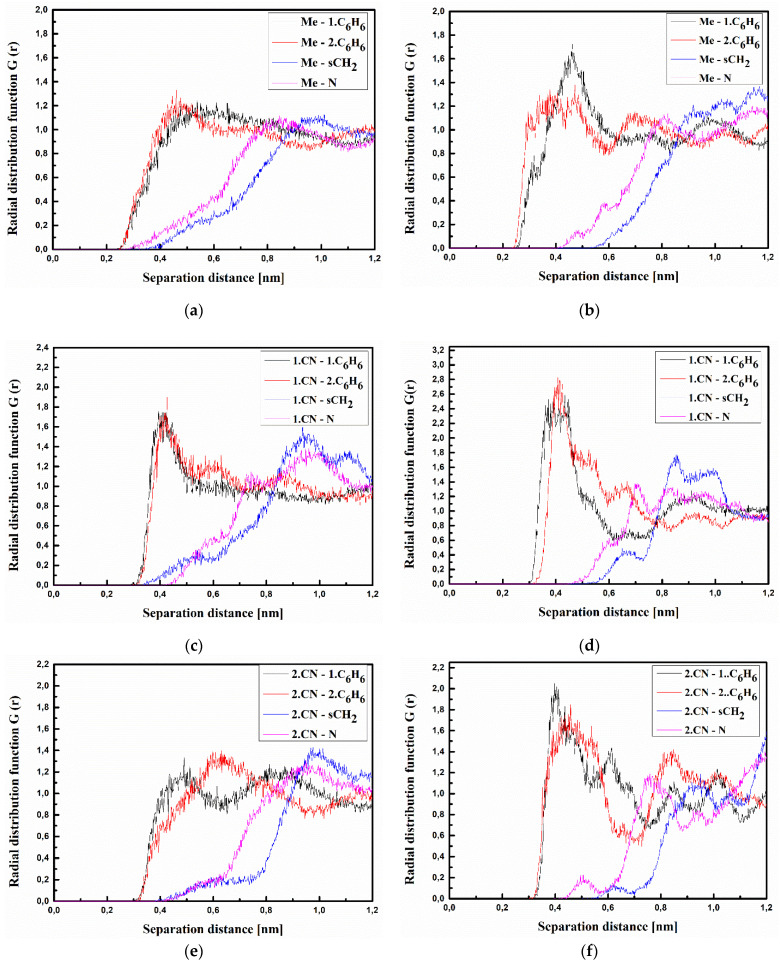
Partial RDFs calculated for distances between the center of mass of different moieties of the A molecule and different subunits of PVK at T = 500 K (**a**,**c**,**e**) and T = 300 K (**b**,**d**,**f**) for the bulk system. The RDFs are presented for the methyl group (Me) of the A molecule and different subunits of PVK mer (**a**,**b**); for cyanovinyl CN1 at the side of the molecule A and different subunits of PVK mer (**c**,**d**); and cyanovinyl CN2 at the back of the molecule A and different subunits of PVK mer (**e**,**f**).

**Figure 4:**
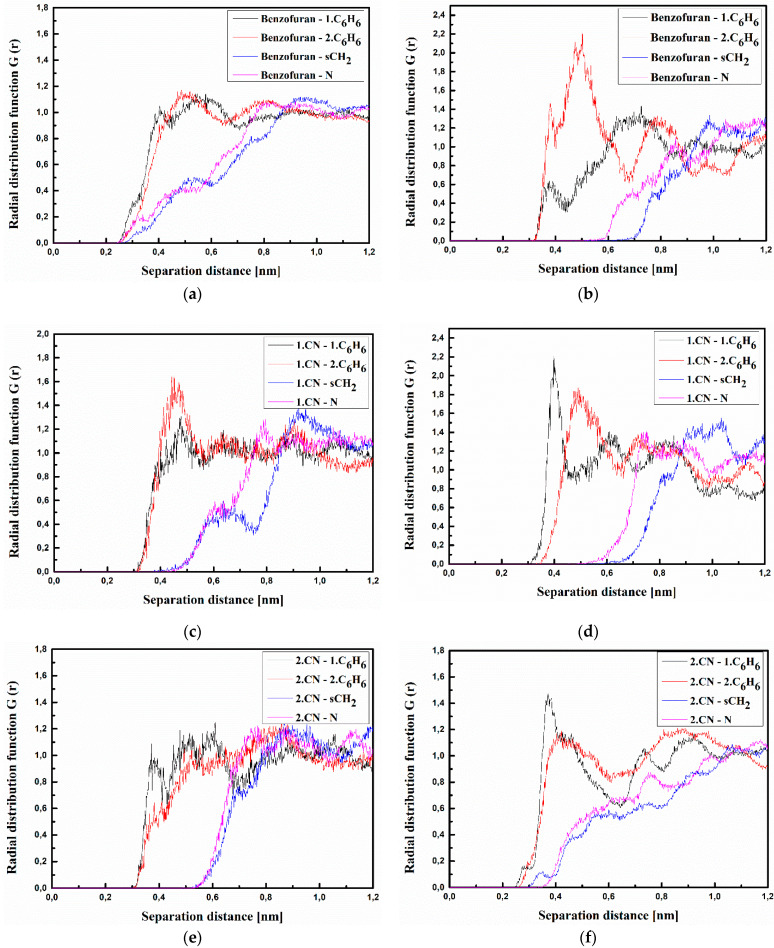
Partial RDFs calculated for distances between the center of mass of different moieties of the B molecule and different subunits of PVK at T = 500 K (**a**,**c**,**e**) and T = 300 K (**b**,**d**,**f**) for the bulk system. The RDFs are presented for the benzofuran group of the B molecule and different subunits of PVK mer (**a**,**b**); for cyanovinyl CN1 at the side of the molecule B and different subunits of PVK mer (**c**,**d**), and cyanovinyl CN2 at the back of the molecule B and different subunits of PVK mer (**e**,**f**).

**Figure 5:**
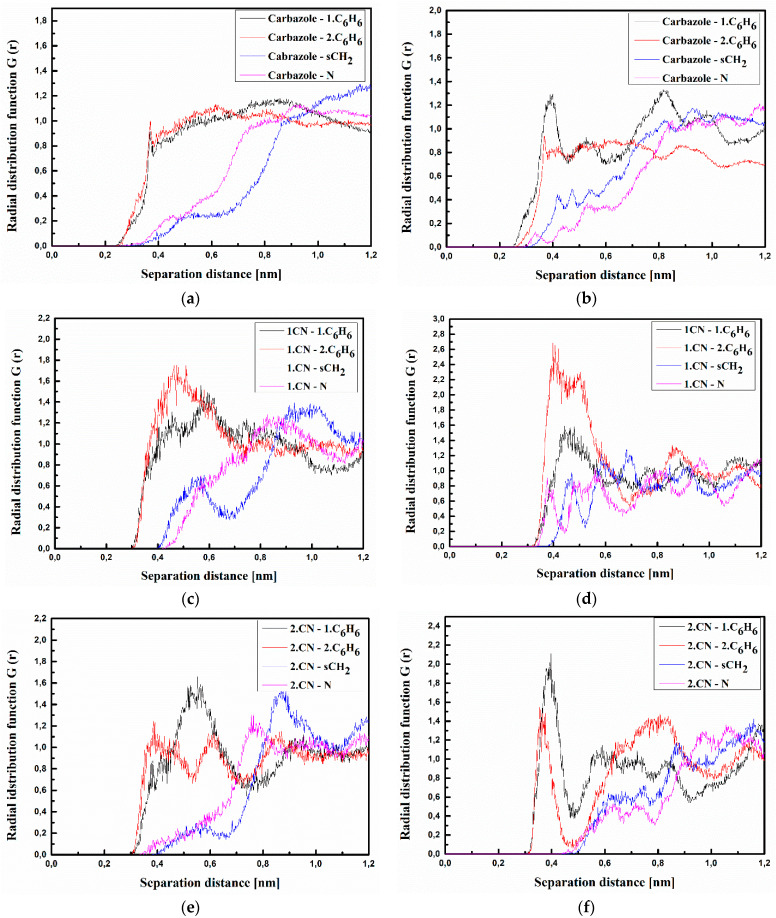
Partial RDFs calculated for distances between the center of mass of different moieties of the C molecule and different subunits of PVK at T = 500 K (**a**,**c**,**e**) and T = 300 K (**b**,**d**,**f**) for the bulk system. The RDFs are presented for carbazole group of C molecule and different subunits of PVK mer (**a**,**b**); for cyanovinyl CN1 at the side of the molecule C and different subunits of PVK mer (**c**,**d**); and cyanovinyl CN2 at the back of the molecule C and different subunits of PVK mer (**e**,**f**).

**Figure 6:**
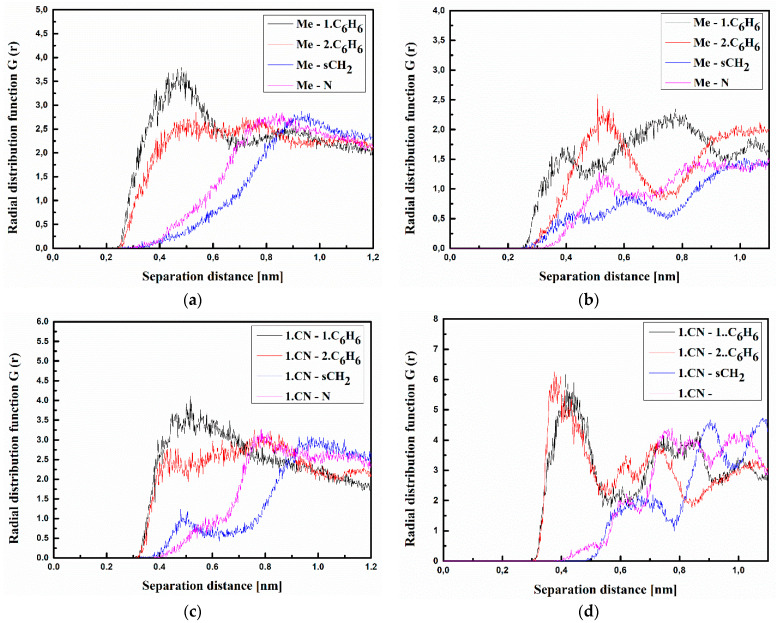

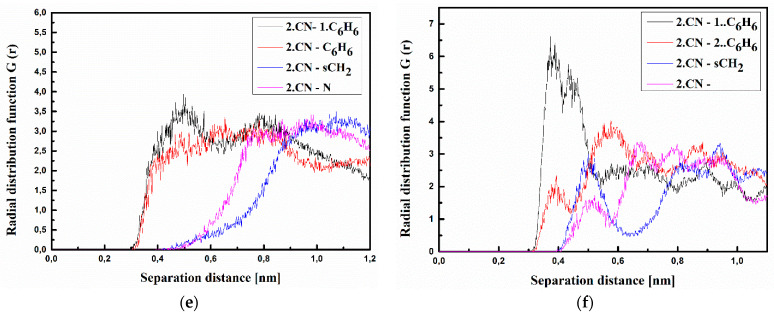
Partial RDFs calculated for distances between the center of mass of different moieties of the A molecule and different subunits of PVK at T = 500 K (**a**,**c**,**e**) and T = 300 K (**b**,**d**,**f**) for the thin film composite system. The RDFs are presented for methyl group (Me) of A molecule and different subunits of PVK mer (**a**,**b**); for cyanovinyl CN1 at the side of the molecule A and different subunits of PVK mer (**c**,**d**); and cyanovinyl CN2 at the back of the molecule A and different subunits of PVK mer (**e**,**f**).

**Figure 7:**
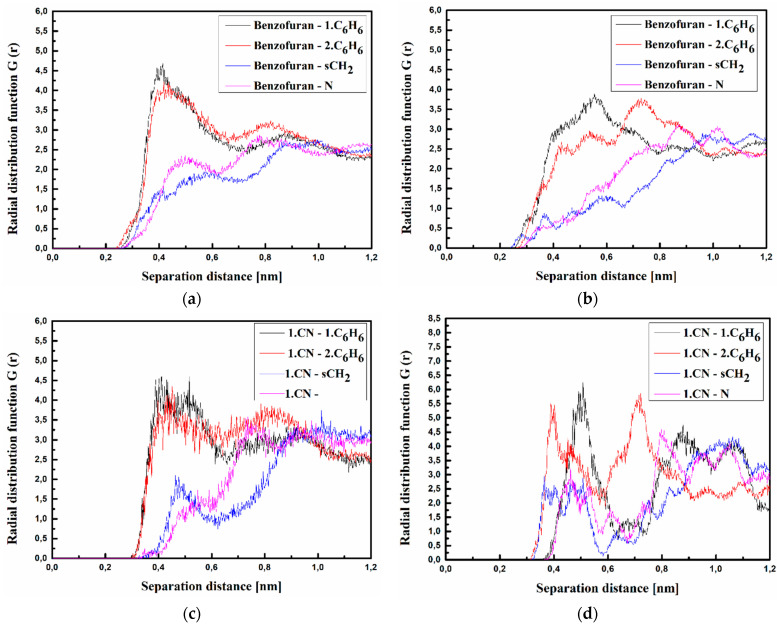

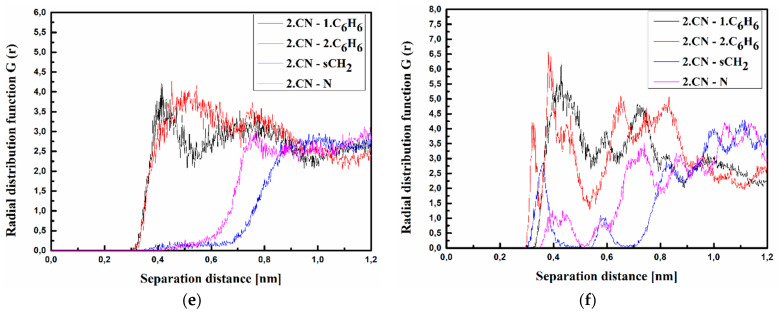
Partial RDFs calculated for distances between the center of mass of different moieties of the B molecule and different subunits of PVK at T = 500 K (**a**,**c**,**e**) and T = 300 K (**b**,**d**,**f**) for the thin-film system. The RDFs are presented for benzofuran group of B molecule and different subunits of PVK mer (**a**,**b**); for cyanovinyl CN1 at the side of the molecule B and different subunits of PVK mer (**c**,**d**); and cyanovinyl CN2 at the back of the molecule B and different subunits of PVK mer (**e**,**f**).

**Figure 8:**
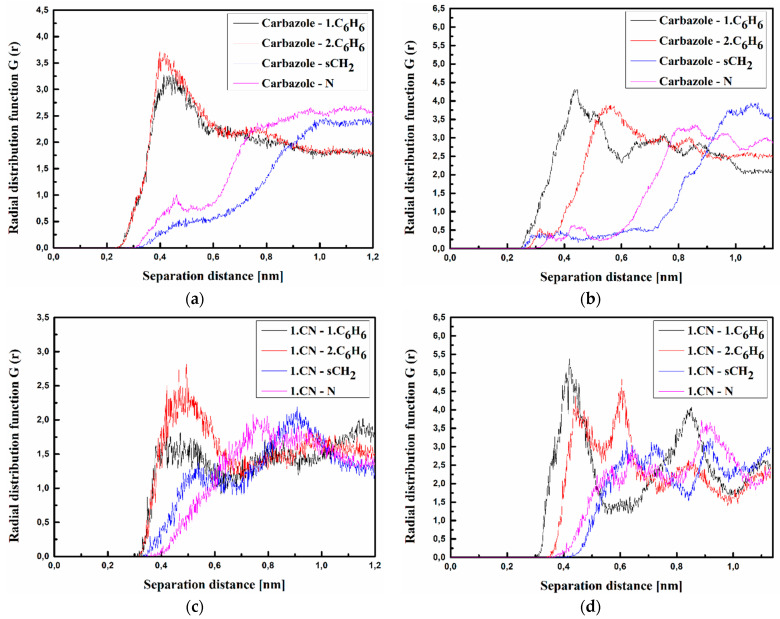

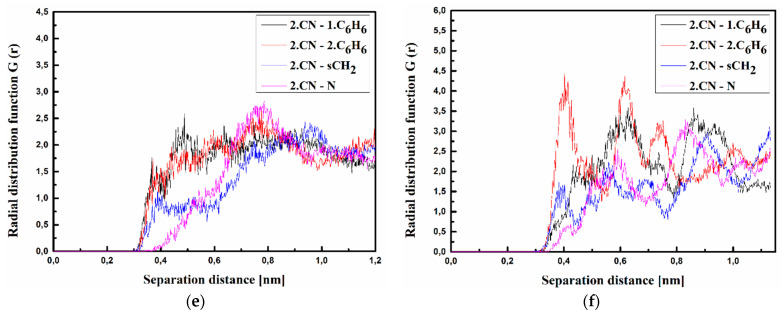
Partial RDFs calculated for distances between the center of mass of different moieties of the C molecule and different subunits of PVK at T = 500 K (**a**,**c**,**e**) and T = 300 K (**b**,**d**,**f**) for the thin film system. The RDFs are presented for carbazole group of C molecule and different subunits of PVK mer (**a**,**b**); for cyanovinyl CN1 at the side of the molecule C and different subunits of PVK mer (**c**,**d**); and cyanovinyl CN2 at the back of the molecule C and different subunits of PVK mer (**e**,**f**).

The calculations of RDFs were based on the expression:

(2) $$g_{AB}\left(r\right)=\frac{\left\langle \rho_{B}\left(r\right)\right\rangle }{{\left\langle \rho_{B}\right\rangle }_{loc}}=\frac{1}{{\left\langle \rho_{B}\right\rangle }_{loc}}\frac{1}{N_{A}}\overset{N_{A}}{\underset{i\;\in \;A}{\sum }}\overset{N_{B}}{\underset{j\;\in B}{\sum }}\frac{\delta \left(r_{ij}-r\right)}{4\pi r^{2}}$$

where <*ρ_B_*(*r*)> is the density of group B at distance r around group A, and <*ρ_B_*>*_loc_* is the density of group B averaged over the sphere around group A with the highest value of distance between particles *r_max_*, which is equal to half the unit cell length. The RDFs calculated for the investigated structures at T = 500 K and T = 300 K are presented in Figure 3, Figure 4 and Figure 5 for bulk composite systems, and Figure 6, Figure 7 and Figure 8 show the RDF for thin films.

Analyzing the data presented in Figure 3, one can see that the A molecule is located close to the carbazole group of the PVK polymer chain. The closest distance between all defined groups of molecule A (both CN groups and Me group) is equal to 0.4 nm for the system in the liquid phase (at 500 K) (see Figure 3a,c,e). The distance between Me groups of the A molecule and both C_6_H_6_ groups of the PVK is going to be shorter (0.3 nm) when the A/PVK system is in a glassy state (at 300 K) (see Figure 3b). This allows us to conclude that in the A/PVK composite, the polymer is rolled up such that its carbazole groups remain outside.

The same situation is observed for the B/PVK system (see Figure 4). Here, one can see that molecule B even in a liquid state (see Figure 4a,c,e) is closer to the polymer chain than molecule A. The distance between the benzofuran group of molecule B and both C_6_H_6_ groups of PVK is equal to 0.3 nm (Figure 4a). This means that these two flat groups assume a parallel arrangement to one another. In the glassy state, the distance between the 2.CN group of the B molecule and the C_6_H_6_ groups of PVK is equal to 0.3 nm (see Figure 4f). In addition, the distance between the 2.CN group and sCH_2_ becomes shorter (0.35 nm) compared with the liquid state (see Figure 4f). This means that in the glassy state, the molecule B goes deeper into the structure of the twisted polymer.

Molecule **C** is also located at a distance of 0.4 nm from the side groups of the polymer chain (see Figure 5). In addition, here, the polymer is also twisted, keeping the carbazole group outside the twisted chain. In the liquid state (see Figure 5a), the carbazole group of molecule **C** is located at the closest distance to the polymer. The RDF peak corresponding to the carbazole-1.C_6_H_6_ or carbazole-2.C_6_H_6_ distance is more spread than what is observed for the other RDF functions (Figure 5a). Molecule **C** enters deeper into the polymer in the glassy state (Figure 5b,d,f). This is seen from the emerging peaks characterizing the Carbazole-N distance (see Figure 5f).

The RDFs calculated for the thin films of the A/PVK, B/PVK, and C/PVK composites are presented in Figure 6, Figure 7 and Figure 8. One may also see that, here, the distance between A, B, and C molecule and PVK is equal to 0.4 nm in the liquid state (see Figure 6, Figure 7 and Figure 8 labeled as a, c and d). Chromophores are located close to the carbazole moiety of the PVK polymer. One can conclude that the character of the RDFs of the thin films in a liquid state (see Figure 6, Figure 7 and Figure 8 labeled as a, c and d) are similar to the one of the bulk system (see Figure 3, Figure 4 and Figure 5 labeled as a, c and d). The first RDFs peaks observed for the A/PVK composite are wider than what was observed for the bulk system (see Figure 6). This proves that the surface of the system is less dense than the interior. As was observed for the bulk form of the B/PVK system also in the glassy state of the B/PVK thin film, the B molecule is going to be deeper inside the polymer chain. The significant peak characterizing the 2.CN–sCH_2_ distance proves this statement (see Figure 7e). The presented data allow us to conclude that the PVK has exposed sCH_2_ groups when twisting the polymer chain. The C molecule of the C/PVK thin film in the liquid state is located close to the carbazole group of the polymer (see Figure 8a). In the glassy state of the C/PVK thin film, the carbazole group of C is going to be close to the sCH_2_ and N groups of the PVK (see Figure 7b). Concluding, one can say that the close distance between PVK and chromophores appearing in B/PVK and C/PVK systems can affect the optical properties of the composites.

### 3.2. Reorientation of Chromophores

**Figure 9:**
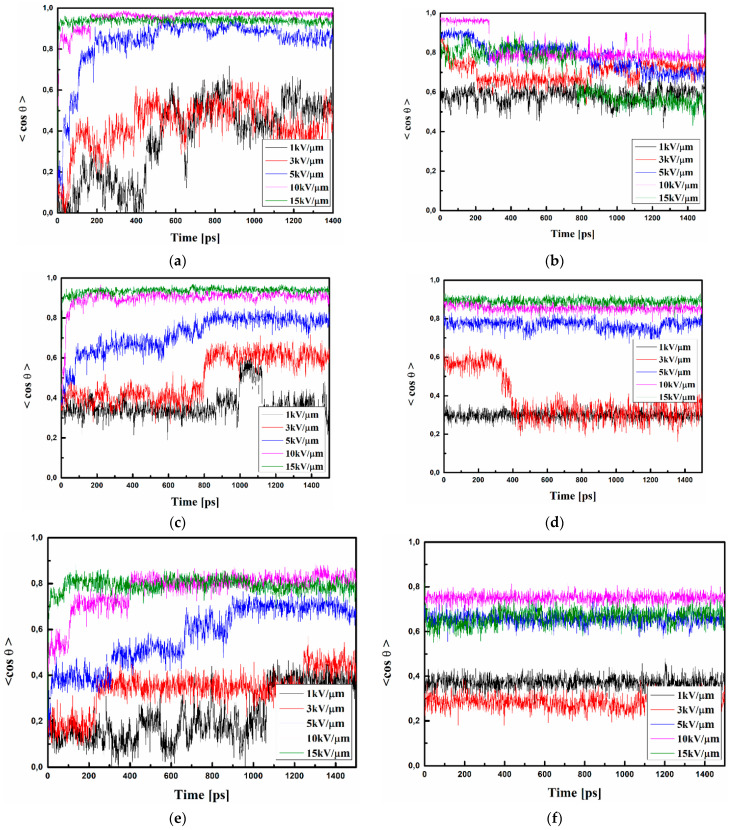
Changes in the value of the order parameter <*cosθ*(*t*)*>* versus the time of simulation and applied external electric field calculated by the MD technique for the A/PVK (**a**,**b**), B/PVK (**c**,**d**), and C/PVK (**e**,**f**) composites in the volumetric form at the temperature of 500 K (**a**,**c**,**e**) and glassy state (300 K) after the simulated annealing (**b**,**d**,**f**).

**Figure 10:**
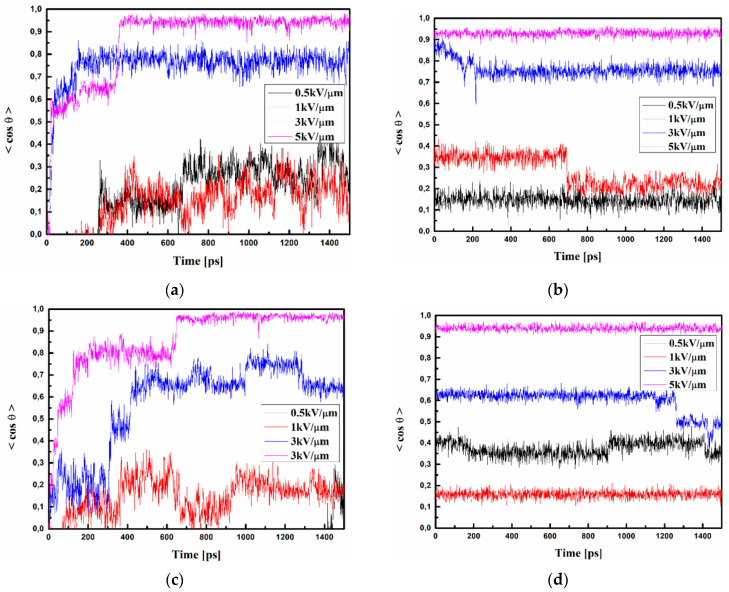

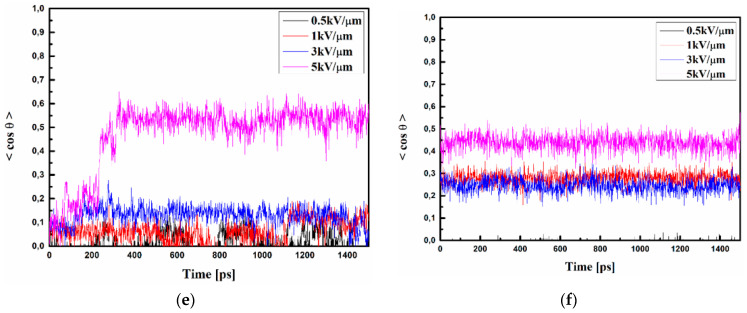
Changes in the value of the order parameter <*cosθ*(*t*)*>* versus the time of simulation and applied external electric field calculated by MD technique for the A/PVK (**a**,**b**), B/PVK (**c**,**d**), and C/PVK (**e**,**f**) composites in the thin film form at the temperature of 500 K (**a**,**c**,**e**) and glassy state (300 K) after the simulated annealing (**b**,**d**,**f**).

The external electric field-induced reorientation of the chromophores was investigated by computing the <*cosθ*(*t*)*>* based on the time-dependent angle between the electric dipole moment of the chromophore and the direction of the external electric field (see Figure 1). The degree of alignment depends on the electric field strength and the spatial extension of the dipolar molecules. The values of <*cosθ*(*t*)*>* versus time of simulation and external electric field intensity are presented in Figure 9 and Figure 10 for volumetric and thin film forms, respectively. Presented data were computed by averaging over modeled equivalent structures each of the three composites A/PVK, B/PVK, and C/PVK. The external electric field was applied for the A/PVK, B/PVK, and C/PVK composites in bulk form when they were in the liquid state at 500 K. Then, the investigated composites were modeled by SA to be in a glassy state (at 300 K). The SA was performed with the presence of an external electric field.

The A/PVK composite in bulk form poled by the external electric field equal to 15, 10, and 5 kV/μm has almost completely aligned chromophores (see Figure 9a). The flexibility of the A/PVK composite is significant and an electric field equal to 1 or 3 kV/μm aligns the A molecules with 40% (see Figure 9a). The SA stabilizes an alignment of the chromophores from 60% up to 80% (see Figure 9b). It is important to notice that chromophores aligned at 500 K by the 1 and 3 kV/μm fields were further tidied up during the SA.

The B/PVK composite exhibits a very satisfactory alignment of the system, applying an external electric field equal to 15, 10, and 5 kV/μm (Figure 9c). These electric fields align chromophores B with 80–95%. Lower electric fields equal to 1 and 3 kV/μm align chromophores B with 40% and 60%, respectively. The SA keeps the alignment of the chromophores treated by 15, 10, and 5 kV/μm electric fields at the level 80–90%, but the lower electric field is too small to keep the alignment of the B chromophores at the appropriate level (see Figure 9d). The alignment of B chromophores decreases up to 30% in the liquid state (at 300 K).

One may see that composite material C/PVK achieved a slightly lower degree of alignment in comparison to the A/PVK and B/PVK systems. The order parameter <*cosθ*(*t*)*>* calculated for *C*/PVK in the glassy state oscillates around the value 70% for the system aligned by the electric field equal to 5, 10, or 15 kV/μm (see Figure 9e). The C/PVK composites with applied electric fields equal to 3 or 1 kV/μm achieved an order parameter below 40%. The SA solidification of the C/PVK does not significantly change the poling alignment of the C molecules compared to the system in a glassy state (see Figure 9f). One may conclude that the C molecules are inferior to order them but, also, they do not reorient during SA. This is due to the size of molecule C, which is larger than molecule A or B.

The same procedures of pooling simulations were implemented for thin films of the A/PVK, B/PVK, and C/PVK composites, and the results are presented in Figure 10. The used external electric field, equal to 0.5, 1, 3, or 5 kV/μm, is lower than the one used for bulk materials. The A/PVK thin film shows that when using an external electric field of 5 kV/μm, the degree of chromophore arrangement is set at 95% (see Figure 10a) and, for an electric field equal to 3 kV/μm, this is around 80%. During controlled cooling, their order parameters dropped slightly to 93% for an external electric field equal to 5 kV/μm and the value of 75% for 3 kV/μm (see Figure 10b). The order parameter achieved for a thin film of A/PVK with an external electric field equal to 0.5 and 1 kV/μm is fixed at the level of 15% and 20%, respectively.

Regarding the B/PVK thin film, one may see that when using an external electric field of 5 kV/μm, the orientation of the chromophores is achieved at the level of 95% (see Figure 10c). Additionally, this order parameter is also maintained after the SA procedure (Figure 10d). The B molecules aligned by the electric field equal to 3 kV/μm obtain an order parameter of 60%, but the alignment is not stable during the time and decreases after the SA process (see Figure 10c,d, respectively). Using an electric field equal to 1 or 0.5 kV/μm, an insufficient structure arrangement was achieved.

The order parameters of C/PVK thin films poled by an external electric field with intensity of 1, 3, or 5 kV/μm is achieved at levels of 10, 15, and 50%, respectively (see Figure 10e). Simulated cooling does not decrease the achieved order parameters (see Figure 10f). The thin film of the C/PVK is difficult to be poled but, instead, it is also stable during the cooling and solidification procedure. One may see that the C/PVK thin film is the most difficult system to achieve a high degree of chromophores arrangement compared to the bulk material. This could be due to the appearance of intermolecular interactions between polymer and chromophores lying close one to the other as well as due to the size of the C chromophore.

### 3.3. Electric and Optical Properties of Chromophores

The electric and optical properties of the A, B, and C molecules were calculated in a vacuum and PVK polymer environment using the TDDFT method, applying the GGA/B3LYP functional. All obtained results are collected in Table 2. The properties of the molecules for which calculations were carried out in vacuum correspond to the values described as *F_x_*,*F_y_*,*F_z_* = 0,0,0. The data obtained for the A, B, and C molecules in PVK polymer are denoted as *F_x_*,*F_y_*,*F_z_* ≠ 0,0,0. The benzonitrile group present in all investigated molecules (see Figure 2) works as the acceptor moiety attached to the donor part of chromophores through the cyanovinyl chain. In the case of two molecules (**A** and **B**), the donor is represented by the methylfuran and benzofuran moiety, respectively, and the **C** molecule is composed of the carbazole group. These different donor moieties of the investigated molecules influence their structural and electronic properties. The structural properties of the A, B, and C molecules are described in our previous work [13]. The electronic properties of the A, B, and C molecules in PVK were calculated based on the structures of composites in the volumetric form obtained from MD simulations. The structures taken into account are these modeled at 300 K with a removed poling external electric field. This means that the chromophores located in the polymer matrix were firstly aligned in the liquid state; then, the systems were cooled up to a glassy state, keeping the external electric field acting, and then the electric field was switched off and the system was relaxed for a time equal to 1.5 ns. The last structure obtained from the described simulation was taken to calculate the intensity of the local electric field acting on the chromophore according to the theory of local field approximation described in our previous work [32]. In the framework of local field theory, linear and nonlinear macroscopic susceptibilities are related to molecular properties by local field factors, which describe the effect of the electric field (*F*) on a molecular site induced by the intermolecular interaction. In discrete local field theory, the *F* is computed by considering the molecular environment rigorously, without resorting to a continuum or mean field approximations.

Analyzing the data collected in Table 2, one can see that the most polar is molecule A, whose electric dipole moment is equal to 7.48 D. One may see that the A and B molecules possess a similar *ΔE_HOMO-LUMO_*. The carbazole moiety twisting the molecular structure significantly decreases the *ΔE_HOMO-LUMO_* of the molecule **C** compared with the A and B chromophores, reducing its electric dipole moment (*μ* = 5.02 D).

The electric properties were also calculated for the chromophores embedded into the polymer matrix. In this case, the local electric field *F* acting on the chromophores was calculated for each relaxed structure coming from the MD simulations. The obtained data are presented in Table 2. The highest local electric field (*F* = 3.82 GV/m) is acting on the C molecules in the Z-direction of the laboratory coordinate system. It is also the direction of the electric dipole moment of the chromophore C. The local electric field acting on A and B molecules is equal to 2.56 and 1.85 GV/m, respectively. One may see that the high intensity of the local electric field acting on molecule C significantly changes its electric properties. The important changes are seen in the increase in electric dipole moment from 5.02 D up to 12.39 D as well as in the decrease in *ΔE_HOMO-LUMO_* from 8.45 eV up to 5.65 eV. The NLO properties of the molecules are proportional to the dipole moment and inversely proportional to the *HOMO-LUMO* energy gap difference. In this case, molecule **C** embedded into the PVK matrix should be favorable for the NLO applications.

In Table 2, static and frequency-dependent polarizabilities ($\lambda =\frac{2\pi c}{\omega }=1064\;\mathrm{nm}$) calculated for A, B, and C molecules in a vacuum, and PVK in the volumetric form, are presented. Here, the selected components of the second- and third-order hyperpolarizabilities are also collected. It should be mentioned that all molecules were rotated to have a static electric dipole moment along the *Z*-axis of the laboratory coordinate system.

The average value of polarizability (*α_av_*) has the highest value for the C molecule. This is true for the static and frequency-dependent parameters. The acting local electric field does not change the values of the calculated polarizabilities for the A and B molecules, but it does increase the static and frequency-dependent polarizabilities of the **C** molecules. The hyperpolarizability *β_vec_* has the highest values for the B molecule. The static values of *β_vec_* for the A and C molecules are significantly smaller. This difference is not so important for frequency-dependent *β_vec_* for the A molecule. The highest value of the static *γ_vec_* was obtained for molecule C with a little bit lower value for the B molecule. For the frequency-dependent *γ_vec_*, the B molecule is better than for the molecules C and A. The local electric field drastically increases the *β_vec_* for the C molecule, decreasing the mentioned parameter for the B molecule in the static and frequency-dependent regime. The local electric field almost does not change the *β_vec_* for the A molecule. Important changes are seen for the B molecule for the parameters possessing components in the Z-direction (*β_z_* and *β_zzz_*). The second-order hyperpolarizabilities of the investigated chromophores are also affected by the local electric field. The static *γ_vec_* decreases with the local electric field for the A and B molecules and slightly increases for the B molecule. However, the frequency-dependent *γ_vec_* of the C molecule increases significantly in the PVK environment. The changes observed for the A and B molecules in the PVK environment are not so spectacular.

### 3.4. UV–Vis Absorption Spectra

**Figure 11:**
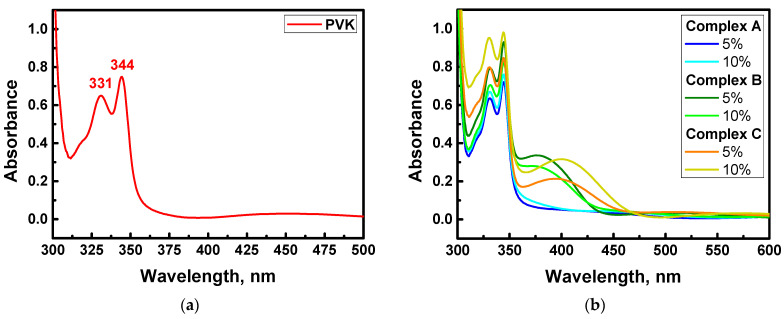
Absorption spectra measured for PVK (**a**) and studied A/PVK-, B/PVK-, and C/PVK-based samples (**b**) in the form of thin films.

The absorption spectra of the A-, B-, and C-based samples were measured in a range of UV–Vis wavelengths (Figure 11). It was noted that at a wavelength corresponding to the generated second harmonic (532 nm), the absorbance is insignificant; however, at 355 nm, which corresponds to the generated third harmonic, absorbance is not negligible. This means that UV–Vis absorption influences the response results. This determines that the generated response is absorbed at the same time. It is necessary to take into account the absorption coefficient in NLO calculations. The calculated values of absorption coefficients for 355 nm (THG) are given in Table 3. The peaks at 331 and 344 nm come from polymer matrix PVK. Spectra presented in Figure 11 correspond with computationally predicted *ΔE_HOMO-LUMO_* collected in Table 2.

### 3.5. NLO Susceptibility

Second-order nonlinear optical susceptibility was calculated by using the Lee model [33]:

(3) $$\chi^{\left(2\right)}=\chi_{Quartz}^{\left(2\right)}\left(\frac{2}{\pi }\right)\left(\frac{L_{Quartz}^{coh}}{d}\right)\sqrt{\frac{I^{2\omega }}{I_{Quartz}^{2\omega }}}$$

where $\chi_{Quartz}^{\left(2\right)}=1\cdot 10^{-12}\;{m\;V}^{-1}$ [34], $L_{Quartz}^{coh}=21\;\mu m$ is the coherence length of the reference material, *d*—the thickness of the sample, and $I^{2\omega }$ and $I_{Quartz}^{2\omega }$ are the SHG intensities of the thin film and reference material, respectively. The calculated susceptibilities for the experimentally obtained data are given in Table 3.

Furthermore, third-order nonlinear optical susceptibility was calculated by using the Kubodera–Kobayashi model [35]:

(4) $$\chi^{\left(3\right)}=\chi_{Silica}^{\left(3\right)}\left(\frac{2}{\pi }\right)\left(\frac{L_{Silica}^{coh}}{d}\right)\left(\frac{\frac{\alpha d}{2}}{1-exp\left(-\frac{\alpha d}{2}\right)}\right)\sqrt{\frac{I^{3\omega }}{I_{Silica}^{3\omega }}}$$

where $\chi_{Silica}^{\left(3\right)}=2\cdot 10^{-22}\;m^{2}V^{-2}$ [36], $L_{Silica}^{coh}=6.7\;\mu m$ is the coherence length of reference material, *α*—linear absorption coefficient, *d*—the thickness of the sample and $I^{3\omega }$ and $I_{Silica}^{3\omega }$ are the THG intensities of the thin film and reference material, respectively. The calculated susceptibilities are given in Table 3.

### 3.6. Second and Third Harmonic Generation

**Figure 12:**
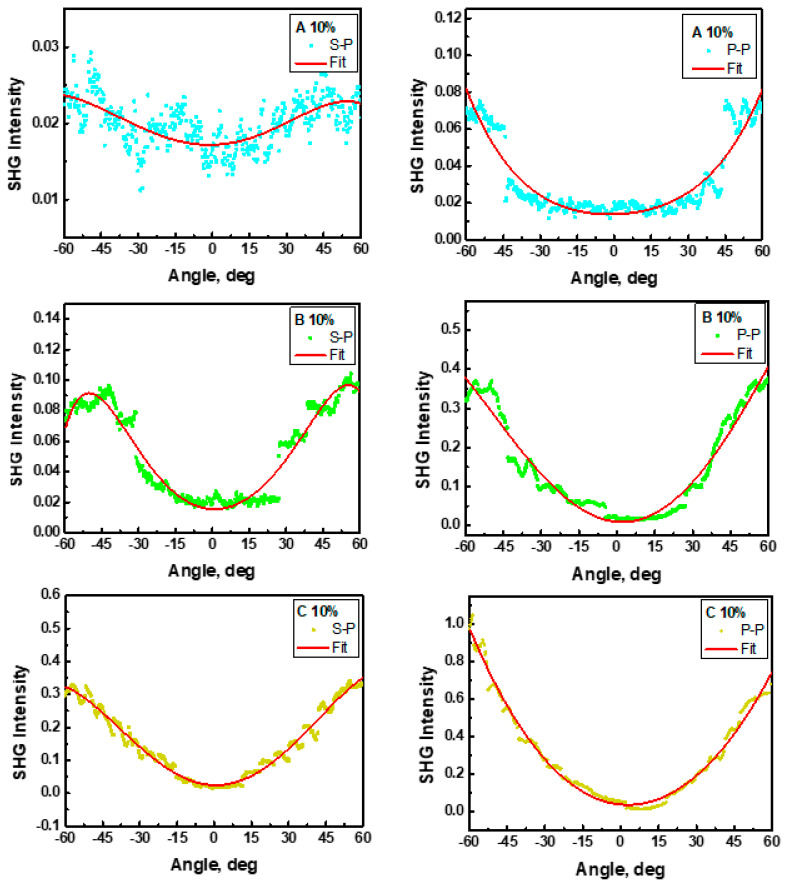
SHG intensity as a function of the incident angle measured for complexes A/PVK, B/PVK, and C/PVK with 10 wt% of A, B, and C chromophores in s-p (**left**) and p-p (**right**) polarization.

Second- and third-order NLO susceptibilities were calculated using theoretical comparative models and then analyzed. Measurements of the SHG response of polymer films with embedded A, B, and C molecules were performed after their poling. The dipole moments of chromophores are oriented perpendicularly to the substrate surface; the maximum interaction between the electric field of the fundamental beam with the dipole moments will be obtained at an angle of *θ* = ± 45°. This targeting will go to zero for the angle of *θ* = 0°. Measurements at two polarization configurations of fundamental and harmonic waves (s-p and p-p) were performed. It was observed that, in the case of SHG, the polarization influences the obtained results. A typical SHG signal exhibits two characteristic bands on either side of the origin for both configurations of polarizations (s-p and p-p) (see Figure 12).

**Figure 13:**
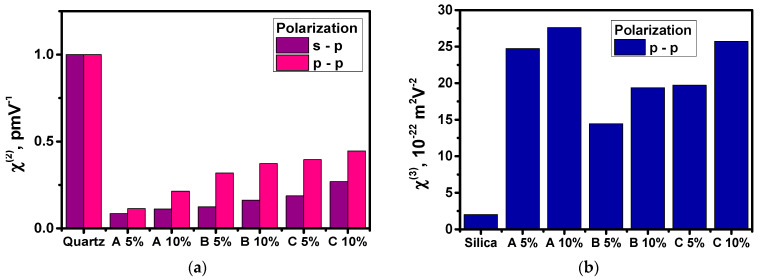
Second- (**a**) and third-order (**b**) NLO susceptibilities calculated for studied A/PVK, B/PVK, and C/PVK thin films with different content of chromophores, taking into account experimentally obtained data.

The experimental SHG response from the A/PVK, B/PVK, and C/PVK films for the p-p input–output configuration of polarization was found to be much higher than that for the s-p polarization. This means that the studied compounds polarize the beam in the vertical direction, which may be noticed from Table 3 and Figure 13. This is also the direction of the electric dipole moment of the chromophores. The obtained SHG results came from the difference in values of *χ*^(2)^ tensor elements responsible for the corresponding scheme of the experiment (*χ*^(2)^*_zzz_* and *χ*^(2)^*_zxx_*). The *χ*^(2)^*_zzz_* and *χ*^(2)^*_zxx_* values obtained for the investigated molecular systems remain in the ratio *χ*^(2)^*_zzz_*/*χ*^(2)^*_zxx_ =* 3 [37]. A lower SHG response has been obtained for the A compound, where a substituent is a methylfuran group. The obtained data agree well with the calculations made. From Table 2, one can see that the lowest value of the *β_zzz_* is obtained for the A molecule. The *β_vec_* calculated for molecules in PVK is a little bit lower for the B molecule than for the A molecule, but the obtained discrepancies may be caused by the too high alignment of the chromophores inside the polymer matrix. The computationally obtained alignment is equal to 90%, which is much higher than in the experimentally observed systems.

Higher second-order NLO susceptibility of 10 wt% C/PVK compared to 10 wt% A/PVK is caused by the presence of a donor carbazole group contributing to better charge transfer occurring in this molecule.

**Figure 14:**
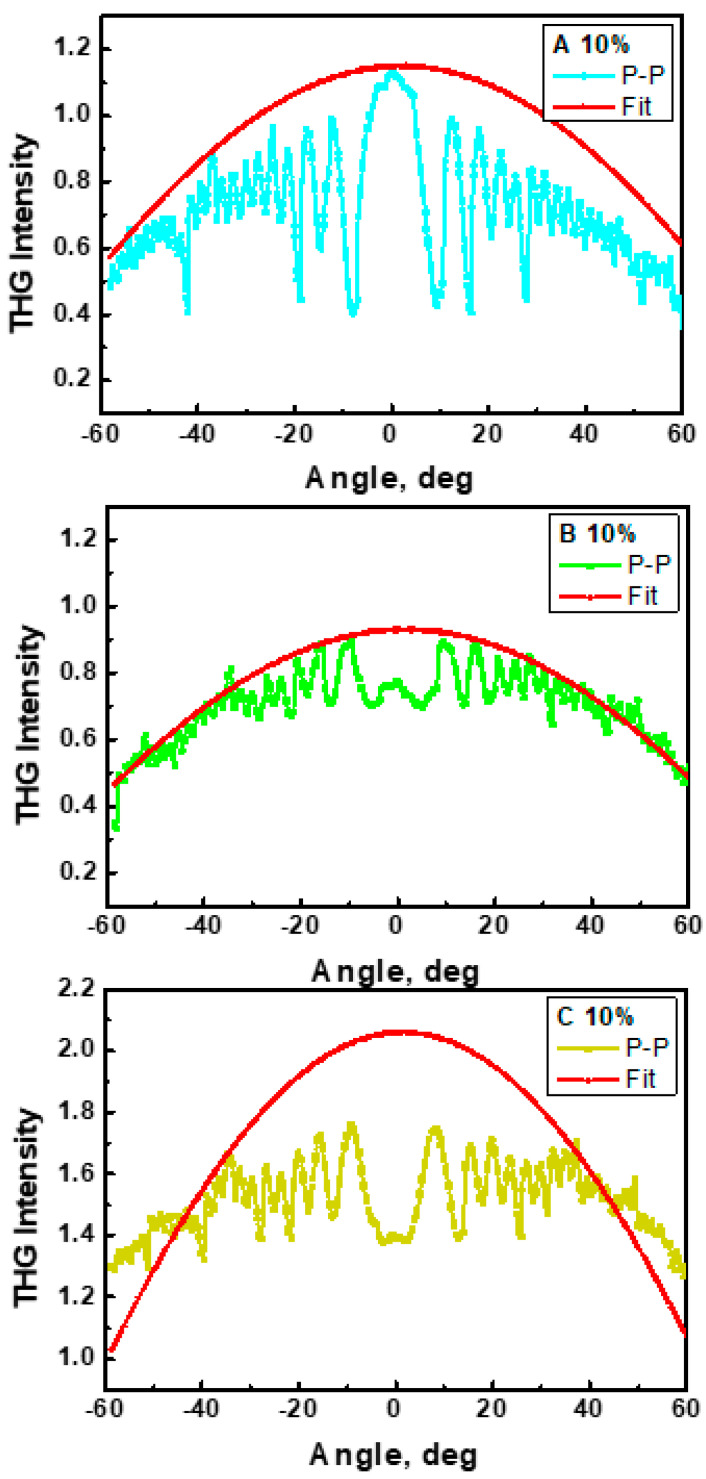
THG intensity as a function of incident angle of A/PVK, B/PVK, and C/PVK composites with 10 wt% of chromophores in p-p polarization.

The third-order NLO properties of the A/PVK, B/PVK, and C/PVK thin films were investigated experimentally using the third harmonic generation technique (THG). In Figure 14, the dependences of the THG signal on the fundamental laser beam incident angle are presented. In the case of THG, it was observed that response did not depend on the polarization of the laser beam. As can be seen, the C/PVK film is characterized by a much higher THG response than the other systems. The obtained data agree well with the performed quantum chemical calculations, taking into account the influence of the polymer matrix on the properties of the chromophores; when applying the local field approach, the frequency-dependent *γ_vec_* of the C molecule grows significantly in the PVK environment (Table 2).

In Table 3 and Figure 13, the obtained values of third-order NLO susceptibility are given. These values were calculated with consideration of the optical absorption coefficient α_355nm_ at the wavelength of the third harmonic. From the THG theory, it is known that only the electronic contribution is included in obtained values of *χ*^(3)^. A higher THG response after taking into account the absorption coefficient has been observed for complexes A/PVK and C/PVK in 10 wt% of solutions.

The obtained values of the second-order nonlinear coefficients for the samples A/PVK, B/PVK, and C/PVK ordered by the external electric field are within the range 0.21 − 0.45 × 10^−12^ pm/V for 10 wt%. They are comparable with the second-order susceptibilities of urea (*χ*^(2)^ = 1.0 × 10^−12^ pm/V [38]), and other similar systems [39,40]. These values are slightly higher compared to the second-order susceptibility of the A/PMMA, B/PMMA, and C/PMMA films with the same concentration (*χ*^(2)^ = 0.28 − 0.36 × 10^−12^ pm/V) [13]. The measured *χ*^(3)^ for investigated materials are in the range of 19.38 − 27.61 × 10^−22^ m^2^ V^−2^ for 10 wt% systems, and are much higher than the respective value of the reference silica material (see Table 3). One can also note that the obtained *χ*^(3)^ value for the A, B, and C molecules integrated into the PVK matrices are higher than the *χ*^(3)^ susceptibility values for A, B, and C in PMMA films with 10 wt% (11.36 − 20.84 × 10^−22^ m^2^ V^−2^) [13].

It can be concluded that higher values of NLO factors, especially of the third-order response, were obtained for the tested A, B, and C compounds incorporated into the PVK matrix compared to the PMMA matrix. These observations agree with theoretical studies showing that the environment of a PVK polymer matrix enhances the hyperpolarizability values. This is especially observed for **C** molecule-based systems.

### 3.7. Application

It is possible to control the unit signal by modulating the density of the carriers, stored thermal energy, or the length of interatomic bonds in designated places of the resonator. This can be carried out in a thermal, electrical, or mechanical way. Optimization of these parameters requires insight into the nature of the physical effects that govern the refractive index of different materials. From the point of using NLO materials in optical modulators [41,42], it is very important to understand their optical properties at the microscopic scale.

Third-order NLO processes allow tuning by changing the refractive index in an entirely optical manner. Materials characterized by strong third-order nonlinearity are commonly used in many fields of science as well as in applications used in everyday life. Since the results presented in this paper are based on the study of the third harmonic generation effect, which has an electronic contribution to nonlinear susceptibility, this means that the third-order effects occurring are based on a change in the nonlinear refractive index. Materials that are characterized by a strong third-order NLO response, through changes in the NLO refractive index, are used, among others, in self-phase modulation, based on the phenomenon of pulse frequency spectrum phase change with the change in NLO refractive index. This phenomenon is commonly used in optical fibers [43].

Computer research and simulations show that NLO effects in different materials depend on their chemical properties. The realization of nonlinear effects requires a non-centrosymmetric distribution of charge inside the material, i.e., molecules must be non-centrosymmetric. Organic materials are compounds that are characterized by high nonlinear susceptibility; for this reason, they have an advantage over inorganic compounds. Crystals of organic compounds such as 2-methyl-4-nitroaniline are up to 59 times more efficient in SHG generation than lithium niobates. This means that we can use—for example as a laser modulator—a much smaller and cheaper material. Reducing the control voltage means that the “switching” frequency can be much higher.

The problem revealed by second-order nonlinearity is the difficulty in the creation of such a material in which there would be such an arrangement of molecules that nonlinearities from individual molecules do not tolerate each other. The materials that are tested in the work are placed in a polymer matrix working as a host (HOST), which holds nonlinear molecules in the right position on the task. Mixed nonlinear molecules with the polymer can be heated to the temperature of vitrification of the polymer (then the molecules have adequate energy for free movement), applying a sufficiently strong solid electric field, which causes the molecules to position themselves along the field force lines. Then, the mixture can be gradually cooled, keeping the external electric field acting on the system. With this technology, an unlimited number of nonlinear samples can be combined with a polymer without the threat of a crystallization process, which can position molecules in an antiparallel way. Such easily synthesized material is ideal for production, e.g., photolithography, thin films, and construction of planar waveguides. This paper presents a simulation of such a process on a time scale. Controlling such a process, during which the centrosymmetric material becomes non-centrosymmetric, allows for dynamic control of the SHG process.

## 4. Conclusions

The linear and NLO properties of hybrid materials based on the (Z)-4-(1-cyano-2-(5-methylfuran-2-yl)vinyl)benzonitrile molecule named as A, the (Z)-4-(2-(benzofuran-2-yl)-1-cyanovinyl)benzonitrile named as B and the (Z)-4-(2-(4-(9H-carbazol-9-yl)phenyl)-1-cyanovinyl)benzonitrile molecule named as C embedded into poly(1-vinylcarbazole) (PVK) polymer matrix were investigated in the volumetric and thin film forms, theoretically and experimentally. The studied molecules (A, B, and C) exhibit quadratic and cubic optical nonlinearities related to different substitutions of the donor groups. It was observed that the benzofuran moiety led to the highest value of *β_vec_* and the carbazole group led to the lowest value of *β_vec_* when the molecules were isolated. The mentioned properties are completely changed when the molecules are in the PVK polymer. One can conclude that the C molecule is compatible with the PVK matrix, which increases the NLO properties of the chromophore. The investigated spatial distribution of the chromophores in the polymer matrix using the intermolecular radial distribution function (RDF) shows that the character of the RDFs of the thin films is similar to the character of the bulk system, and this is evidence that the surface of the system is less dense than the interior. The presented data allow us to conclude that the C molecule of the C/PVK thin film in the liquid state is located close to the carbazole group of the polymer, whereas the B molecule is going to be deeper inside the polymer chain, which can influence the optical properties of the composites. The obtained results indicate that the NLO properties of the material are influenced by both the value of the appropriate hyperpolarizability and the tendency to organize chromophores in a polymer matrix under the influence of an external electric field. Investigation of the degree of alignment depending on the electric field strength showed that the composite material C/PVK achieved a slightly lower degree of alignment in comparison to the A/PVK and B/PVK systems. One may conclude that the C molecules are worse in terms of alignment than particles A and B but, also, they do not reorient during simulated annealing, which is connected with the larger size of this chromophore. In addition, the SA process increases the alignment of the C chromophores in thin film structures, which is not observed for the A/PVK and B/PVK systems. The degree of ordering of the patterns in the polymer matrix is of great importance for the second-order nonlinear properties.

The highest THG response was observed for the 10 wt% complex of C/PVK, where the greatest impact on the obtained values was the local electric field, which drastically increased the *β_vec_* for this chromophore. A higher THG response has been observed for complexes based on A and C in 10 wt% solutions. The values of the factors of *χ*^(2)^, and *χ*^(3)^ for C are related to the presence of a strong carbazole donor group and also an influence of polymer matrix on the properties of the chromophores when applying the local field approach. It can be concluded that the C/PVK composite is the most stable structure over time in terms of chromophore ordering and can be used as potential materials for NLO device applications.

## Figures and Tables

**Table 1:** Description of experimentally investigated samples.

Sample	Chemical Structure	Thin Film
Complex A 5 wt%	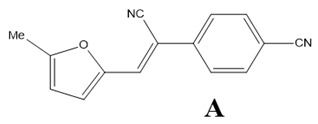	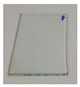
Complex A 10 wt%		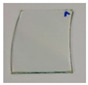
Complex B 5 wt%	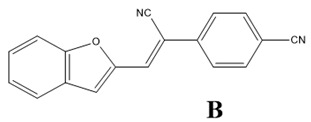	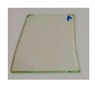
Complex B 10 wt%		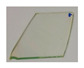
Complex C 5 wt%	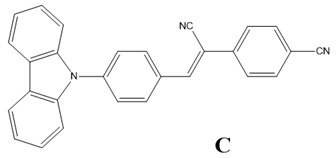	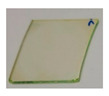
Complex C 10 wt%		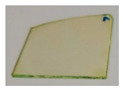

**Table 2:** Electric and optical properties of the A, B, and C molecules calculated in vacuum (*F_x_*,*F_y_*,*F_z_* = 0,0,0) and in the PVK polymer environment (*F_x_*,*F_y_*,*F_z_* ≠ 0,0,0) in volumetric form by using the TDDFT method applying the GGA/B3LYP functional.

Molecule	A		B		C	
Electric field *F_x_*,*F_y_*,*F_z_* [GV/m]	0, 0, 0	1.62, 1.86, 0.69	0, 0, 0	0.91, −1.31, −0.93	0, 0, 0	−1.03, −0.16, 3.82
μ [D]	7.48	8.26	6.88	6.20	5.02	12.39
HOMO [eV]	−8.26	−7.14	−8.29	−8.80	−7.90	−5.30
LUMO [eV]	0.72	1.68	0.44	−0.38	0.55	0.35
*ΔE_HOMO-LUMO_* [eV]	8.98	8.82	8.73	8.42	8.45	5.65
λ = ∞ nm						
*α_xx_*	202.37	202.54	125.65	125.76	324.57	330.38
*α_yy_*	103.10	103.19	249.38	249.60	302.48	301.64
*α_zz_*	284.75	283.97	338.22	338.88	422.19	430.97
*α_av_*	196.74	196.57	237.75	238.08	349.75	354.33
*β*_(*z*;*z*,*z*)_	1241.12	1140.79	1204.01	1325.92	577.38	2634.78
*β*_(*y*;*y*,*y*)_	11.75	84.43	−456.22	−552.36	−122.93	−184.25
*β*_(*x*;*x*,*x*)_	258.13	191.98	−27.93	18.48	228.04	886.73
*β*_(*z*)_	839.62	1614.11	1792.10	1953.61	761.32	4080.97
*β*_(*y*)_	161.67	272.13	−1358.69	−1616.80	153.15	506.09
*β*_(*x*)_	1201.90	1032.03	287.80	392.50	691.34	2610.98
*β_vec_*	1475.01	1935.07	2267.26	1164.73	1039.72	4871.10
*γ*_(*z*;*z*,*z*,*z*)_	208,040.85	200,317.58	263,309.47	273,839.92	273,389.68	457,070.81
*γ*_(*y*,*y*,*y*,*y*)_	19,477.92	20,032.40	117,496.68	119,559.23	100,163.57	102,105.10
*γ*_(*x*,*x*,*x*,*x*)_	49,115.54	50,269.74	25,541.68	25,684.65	113,038.68	167,368.97
*γ_vec_*	100,789.57	98,809.38	163,926.96	168,691.04	173,638.70	120,339.68
λ = 1064 nm						
*α_xx_*	205.67	205.85	126.81	126.76	330.32	336.85
*α_yy_*	103.86	103.95	254.61	249.60	307.44	306.58
*α_zz_*	295.62	294.75	351.91	338.88	433.84	444.11
*α_av_*	201.72	201.52	244.44	238.41	357.20	362.51
*β*_(*z*;*z*,*z*)_	2064.29	1888.23	2186.69	2420.72	961.18	4502.21
*β*_(*y*;*y*,*y*)_	13.76	96.30	−695.71	−827.07	−150.87	−230.18
*β*_(*x*;*x*,*x*)_	368.09	290.41	−34.04	19.55	340.76	1465.61
*β*_(*z*)_	2766.83	2581.94	3153.36	3473.87	1279.00	6836.45
*β*_(*y*)_	235.78	359.37	−2210.82	−2596.59	240.71	886.64
*β*_(*x*)_	1789.19	1560.42	473.16	611.72	1056.34	4419.36
*β_vec_*	3303.35	3038.17	3880.11	2387.40	1676.20	8188.65
*γ*_(*z*;*z*,*z*,*z*)_	822,873.92	779,529.69	1,220,822.88	1,295,989.98	836,301.70	2,839,445.09
*γ*_(*y*,*y*,*y*,*y*)_	25,252.81	26,348.88	359,805.25	373,794.63	170,852.50	177,345.01
*γ*_(*x*,*x*,*x*,*x*)_	116,815.30	118,530.66	34,558.06	34,849.19	255,860.36	695,963.78
*γ_vec_*	321,647.34	308,136.41	538,395.40	568,211.27	421,004.85	1,237,584.63

**Table 3:** The thickness of the layers of the tested samples, the absorption coefficients assigned to them, and effective *χ*^(2)^ and *χ*^(3)^ values obtained for A, B, and C films in PVK matrix, taking into account experimentally measure data.

Sample	Thickness [nm]	*α*_355nm_ [10^3^ cm^−1^]	*χ*^(2)^ [pmV^−1^]		*χ*^(3)^ [10^−22^ m^2^V^−2^]
			s-p	p-p	p-p
Quartz	1000	-	1.00		-
Silica			-		2.00
A 5 wt%	360	10.72	0.085 ± 0.028	0.114 ± 0.030	24.75 ± 0.75
A 10 wt%	320	14.09	0.111 ± 0.028	0.214 ± 0.022	27.61 ± 0.93
B 5 wt%	570	16.03	0.124 ± 0.008	0.319 ± 0.008	14.45 ± 0.32
B 0 wt%	410	18.26	0.162 ± 0.012	0.373 ± 0.013	19.38 ± 0.55
C 5 wt%	510	10.25	0.187 ± 0.008	0.396 ± 0.009	19.72 ± 0.45
C 10 wt%	460	16.27	0.269 ± 0.009	0.446 ± 0.012	25.71 ± 0.62

## Data Availability

The data presented in this study are available on request from the corresponding author.
